# Supplementary material for: Elicited Production of Essential Oil with Immunomodulatory Activity in Salvia apiana Microshoot Culture
Source: Molecules. 2025 Feb 10;30(4):815. doi: 10.3390/molecules30040815 (PMC11858325; doi:10.3390/molecules30040815)
Supplement: Supplementary file 1 [file molecules-30-00815-s001.zip › molecules-3428963-supplementary.pdf]

molecules

MDPI

Article

Elicited Production of Essential Oil with Immunomodulatory Activity in *Salvia apiana* Microshoot Culture

Agata Krol [1,*], Adam Kokotkiewicz [1], Bozena Zabiegala [2], Klaudia Ciesielska-Figlon [3], Ewa Bryl [3], Jacek Maciej Witkowski [3,4], Adam Bucinski [5] and Maria Luczkiewicz [1]

[1] Department of Pharmacognosy, Faculty of Pharmacy, Medical University of Gdansk, Gen. J. Hallera Street 107, 80-416 Gdansk, Poland; adam.kokotkiewicz@gumed.edu.pl (A.K.); mlucz@gumed.edu.pl (M.L.)
[2] Department of Analytical Chemistry, Faculty of Chemistry, Gdansk University of Technology, 11/12 Gabriela Narutowicza Street, 80-233 Gdansk, Poland; bozzabie@pg.edu.pl
[3] Department of Pathophysiology, Faculty of Medicine, Medical University of Gdansk, M. Skłodowskiej-Curie 3a Street, 80-211 Gdansk, Poland; klaudia.ciesielska-figlon@gumed.edu.pl (K.C.-F.); ewa.bryl@gumed.edu.pl (E.B.); jacek.witkowski@gumed.edu.pl (J.M.W.)
[4] Department of Embryology, Faculty of Medicine, Medical University of Gdansk, M. Skłodowskiej-Curie 3a Street, 80-211 Gdansk, Poland
[5] Department of Biopharmacy, Faculty of Pharmacy, Collegium Medicum in Bydgoszcz, Nicolaus Copernicus University in Torun, Jagiellonska Street 15, 85-067 Bydgoszcz, Poland; adam.bucinski@cm.umk.pl
* Correspondence: agata.krol@gumed.edu.pl

 https://doi.org/110.3390/molecules30040815

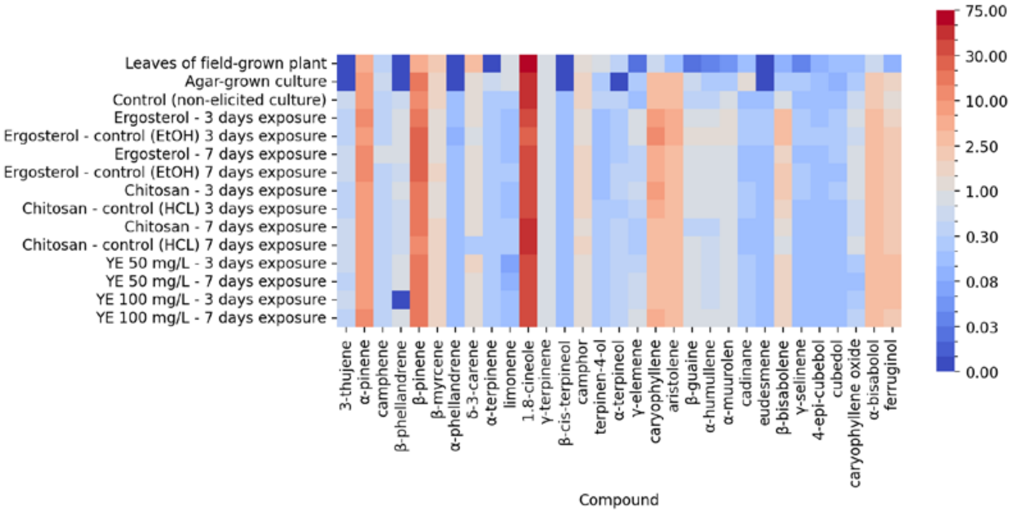

Figure S1. Composition of essential oil samples obtained from leaves of field grown plants and microshoot cultures of *S. apiana*. Heatmap visualization of the data originally presented in Table 1.

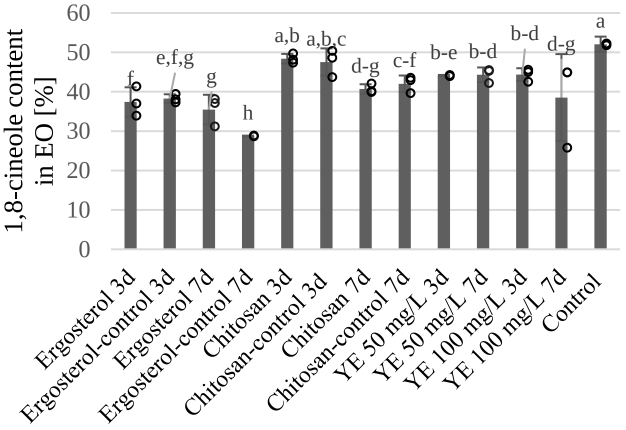

(**a**)

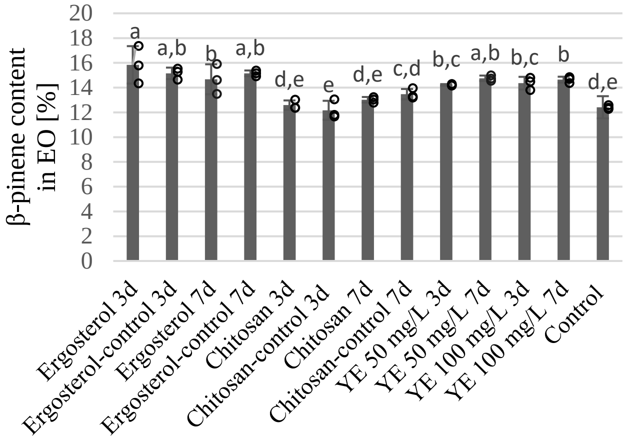

(**b**)

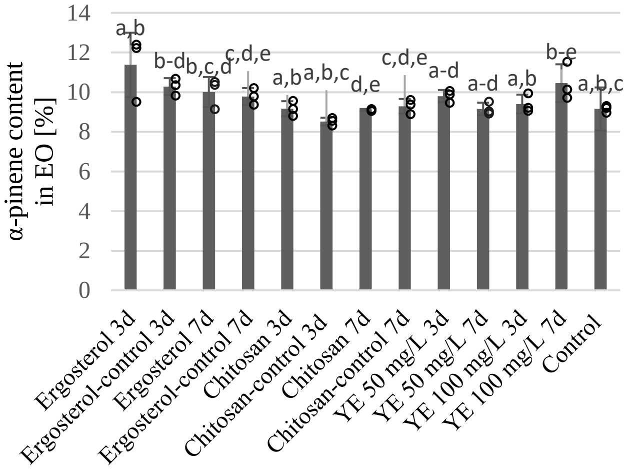

(**c**)

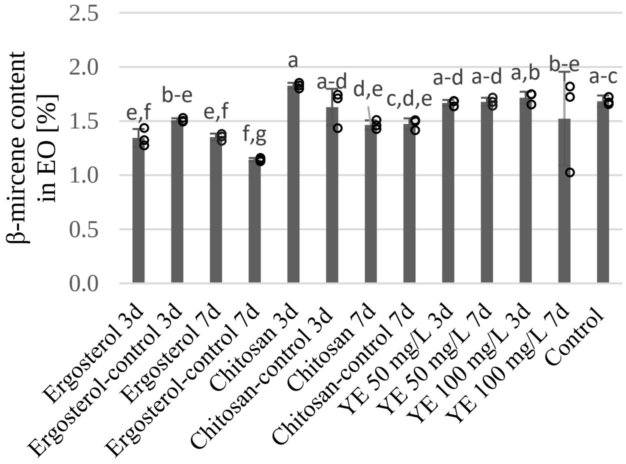

(**d**)

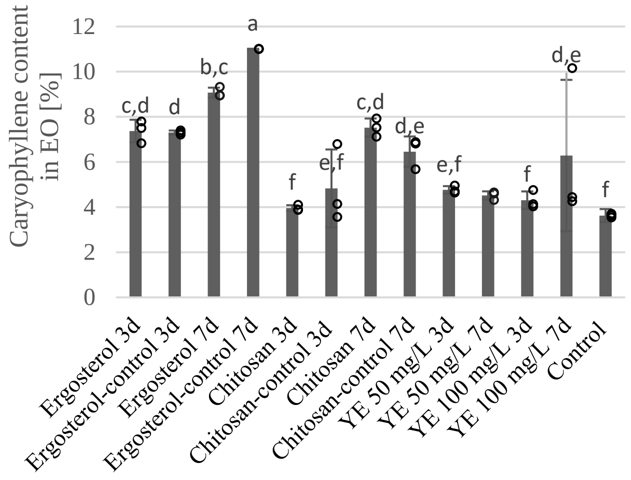

(e)

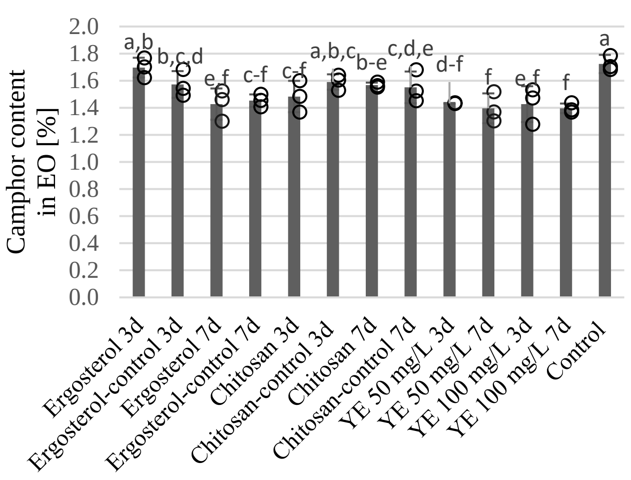

(f)

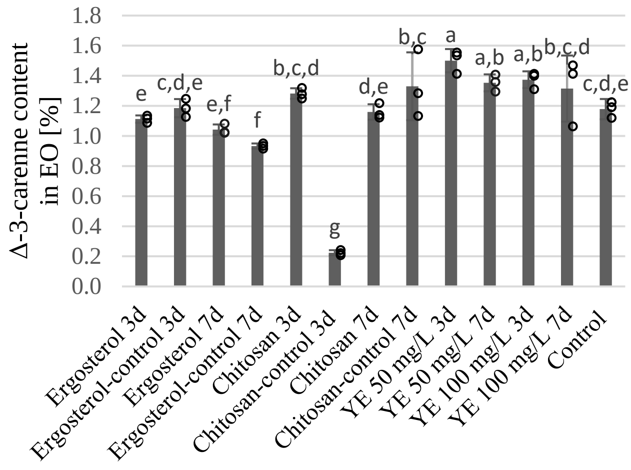

(g)

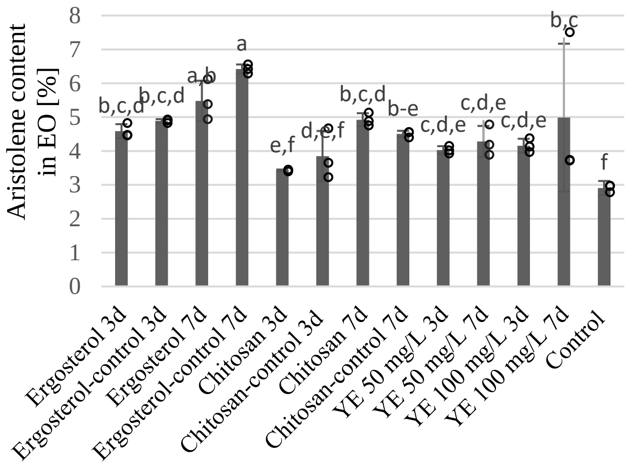

(h)

Figure S2. The effect of elicitation with ergosterol, chitosan and yeast extract (YE) on main volatile terpenoids: 1,8-cineole (a), β-pinene (b), *α*-pinene (c), β-mircene (d), caryophyllene (e), camphor (f), Δ-3-carenne (g), and aristolene (h), in bioreactor-grown microshoots of *S. apiana*. The liquid growth medium was modified by adding elicitors or their respective solvents (positive control) either 3 days (3d) or 7 days (7d) before harvesting. The negative control consisted of biomass cultivated in non-elicited medium for 21 days. The presented values represent arithmetic means from a minimum of six experimental replicates ± standard deviation (SD). Dots represent individual replicates. Different letters indicate significant differences at $p = 0.05$ (one-way ANNOVA followed by Fisher LSD test).
